# Endoscopic stent insertion for anastomotic leakage following oesophagectomy

**DOI:** 10.1308/003588413X13511609956255

**Published:** 2013-01

**Authors:** M Schweigert, N Solymosi, A Dubecz, RJ Stadlhuber, H Muschweck, D Ofner, HJ Stein

**Affiliations:** ^1^Klinikum Nürnberg Nord,Germany; ^2^Szent István University, Budapest,Hungary; ^3^Paracelsus Medical University, Salzburg,Austria

**Keywords:** Esophagectomy, Anastomotic leak, Endoscopic stent implantation, Aortic erosion, Tracheoesophageal fistula

## Abstract

**Introduction:**

Intrathoracic anastomotic leakage following oesophagectomy is a crushing condition. Until recently, surgical re-exploration was the preferred way of dealing with this life threatening complication. However, mortality remained significant. We therefore adopted endoscopic stent implantation as the primary treatment option. The aim of this study was to investigate the feasibility and results of endoscopic stent implantation as well as potential hazards and pitfalls.

**Methods:**

Between January 2004 and December 2011, 292 consecutive patients who underwent an oesophagectomy at a single high volume centre dedicated to oesophageal surgery were included in this retrospective study. Overall, 38 cases with anastomotic leakage were identified and analysed.

**Results:**

A total of 22 patients received endoscopic stent implantation as primary treatment whereas a rethoracotomy was mandatory in 15 cases. There were no significant differences in age, frequency of neoadjuvant therapy or ASA grade between cases with and without a leak. However, patients with a leak were five times more likely to have a fatal outcome (odds ratio: 5.10, 95% confidence interval: 2.06–12.33, *p*<0.001). Stent migration occurred but endoscopic reintervention was feasible. In 17 patients (77%) definite closure and healing of the leak was achieved, and the stent was removed subsequently. Two patients died owing to severe sepsis despite sufficient stent placement. Moreover, stent related aortic erosion with consecutive fatal haemorrhage occurred in three cases.

**Conclusions:**

Stent implantation for intrathoracic oesophageal anastomotic leaks is feasible and compares favourably with surgical re-exploration. It is an easily available, minimally invasive procedure that may reduce leak related mortality. However, it puts the already well-known risk of stent-related vascular erosion on the spot. Awareness of this life threatening complication is therefore mandatory.

Intrathoracic leakage of the oesophagointestinal anastomosis is a devastating complication and one of the major reasons for postoperative mortality following oesophagectomy. Persistent contamination of both the mediastinum and pleural cavity results in mediastinitis, pleural empyema and, eventually, severe sepsis with septic shock. Immediate action is mandatory to avoid a fatal outcome.

The established aims of either surgical or non-operative intervention are prevention of further contamination by closure of the dehiscence and adequate drainage of the leak. Until recently, surgical re-exploration was the preferred way of dealing with this life-threatening situation. However, the results were disappointing and marred by continuous high mortality of between 60% and even 100%.^1,2^ Therefore, conservative treatment has been proposed.^3–5^ The disadvantage of conservative treatment is the ongoing pollution of the mediastinum and pleural cavity through the anastomotic leak. Hence, the reported mortality rate is similar to that of surgical re-exploration and up to 40%.^6^

Endoscopic stent implantation has shown good and reliable results in the treatment of intrathoracic anastomotic leaks in several series throughout the last ten years.^7–10^ Insertion of a self-expanding covered stent is a minimally invasive procedure that provides immediate sealing of the dehiscence. Stent migration and dislocation are the main postinterventional complications and may account for insufficient closure of the leak. Furthermore, stent-related vascular erosion resulting in fatal bleeding has been reported.^11,12^ However, most benefits as well as disadvantages of a new treatment option are only revealed by long-term experience. This study was therefore designed especially to get a long-term perspective on feasibility and results of endoscopic stent implantation as well as the potential hazards and pitfalls.

## Methods

The study included all patients who underwent oesophagectomies at the Department of General and Thoracic Surgery of the Klinikum Nürnberg Nord between January 2004 and December 2011. Our institution is a tertiary referral hospital serving approximately half a million people and providing advanced surgical procedures for an even greater rural and urban population. The department is dedicated particularly to oesophageal surgery and it is one of Germany’s largest high volume centres.

At the beginning of 2004 we adopted endoscopic stent insertion as the primary treatment option for intrathoracic anastomotic leaks. Thus, the study period started in January 2004. A local ethics committee approved this retrospective study and waived the need for individual consent.

### Operative technique

*En bloc* oesophagectomy consisted of an abdominothoracic oesophagectomy with interposition of a pulled up gastric tube and intrathoracic stapled anastomosis above the level of the tracheal carina.^13^ **Transhiatal extended total gastrectomy included resection of the distal oesophagus and intrathoracic stapled oesophagojejunostomy below the level of the tracheal carina.^13^ Furthermore, limited resection of the distal oesophagus with jejunal interposition and a stapled intrathoracic oesophagojejunostomy (Merendino procedure) was carried out.^14^

In general, restoration of the alimentary tract continuity by an intrathoracic anastomosis was preferred. However, some procedures including transmediastinal oesophagectomy as well as transthoracic oesophagectomy with delayed reconstruction required a cervical anastomosis. From 2008 onwards, the operations were performed mainly by two experienced general thoracic surgeons (MS and HJS).

Neoadjuvant therapy was administered as either neoadjuvant chemoradiotherapy for squamous cell carcinoma or neoadjuvant chemotherapy alone for adenocarcinoma of the oesophagogastric junction. The radiation dose was 50.4Gy.

### Diagnosis of anastomotic leak

In cases of a clinically suspected intrathoracic anastomotic leak, endoscopy and computed tomography were carried out immediately. Endoscopy was preferred to radiographic contrast medium swallow for diagnosis of anastomotic leaks because of the possibility of direct visual examination of the anastomosis, quantification of the leak and determining whether the pulled up gastric tube was ischaemic. The endoscopic aspect aids the decision as to whether endoscopic stent insertion is advisable. Additional computed tomography is helpful to rule out advanced pleural empyema or a mediastinal abscess, which would require either percutaneous interventional or surgical drainage.

### Stent insertion

For closure of the anastomotic leak, either a self-expanding, covered metal stent (Ultraflex™, Boston Scientific, Natick, MA, US) or a self-expanding, covered silicone stent (Polyflex^®^, Boston Scientific) was inserted. Stent placement was performed by a gastroenterologist who was well trained in interventional endoscopy. The exact position of the leak was marked on the patient’s skin and the stent was inserted under radioscopy guidance. Correct placement of the stent and successful closure of the leak were always controlled by endoscopy as well as by contrast medium swallow.

### Statistical analysis

Statistical analysis was performed using R statistical software (R Foundation for Statistical Computing, Vienna, Austria). The independence of the studied variable pairs was tested with Fisher’s exact test.

## Results

Overall, 292 consecutive cases were included in this study. There were 243 male and 49 female patients with a mean age of 63.9 years (Table 1). The average American Society of Anesthesiologists (ASA) grade was 2.2 and 74 patients had received neoadjuvant therapy. Surgery was performed mainly for oesophageal malignancies. Squamous cell carcinoma (*n*=96), adenocarcinoma of the oesophagogastric junction (AEG) type I (*n*=127), AEG type II (*n*=45), which is a cancer of the gastric cardia with involvement of the distal oesophagus,^15–17^ and AEG type III (*n*=12), which is a subcardiac gastric carcinoma infiltrating the oesophagogastric junction,^15–17^ were most common.

**Table 1:** Characteristics of patients

Variables	All	Leak	No leak
Total number of patients	292	38	254
Mean age (years)	63.9 (SD: 10.6)	64.8 (SD: 9.1)	63.8 (SD: 10.8) *p*=0.54
Age >75 years	45	6	39 *p*=1.0
Neoadjuvant therapy	74	7	67 *p*=0.36
ASA grade	2.24	2.24	2.21 *p*=1.0
Fatal outcome	33	12 (31%)	21 (8%) ***p*<0.001**

ASA = American Society of Anesthesiologists;

SD = standard deviation

Intrathoracic anastomotic leakage occurred in 38 cases. Endoscopic implantation of either a self-expanding metal or silicone stent was accomplished successfully in 22 patients. Rethoracotomy was mandatory in 15 other cases because of either ischaemia of the pulled up gastric tube or advanced pleural empyema that required surgical debridement. Furthermore, one patient had been managed conservatively without success in the initial year of this study.

### Results of patients with stent insertion

Initial stent insertion was successful in all 22 patients and provided immediate closure and sealing of the leak. Stent migration occurred but endoscopic reintervention was always feasible. Two patients eventually died from septic shock due to severe persisting sepsis despite sufficient stent placement. Moreover, stent-related aortic erosion resulted in fatal haemorrhage in three cases. In those three cases, repeated stent migration and dislocation took place. The insufficient anastomosis was an oesophagojejunostomy in two cases. Postmortem examination confirmed stent-related vascular erosion with formation of an aorto-oesophageal fistula in all three patients.

Fistulisation between the airways and a dehiscent intrathoracic anastomosis occurred in eight cases. It was possible to amend five of these by endoscopic stent insertion. One was managed conservatively and constituted the single patient who received neither stent nor rethoracotomy. Owing to ischaemia of the pulled up gastric tube and necrosis of the airways, rethoracotomy was unavoidable in two patients who both died.

The five patients with a leak induced tracheo-oesophageal fistula received a single oesophageal stent (three cases), a single tracheal stent (one case) or both (one case). Only the patient who received just a tracheal stent died. He was one of the two patients who died from ongoing sepsis despite sufficient stent placement.

In the 14 remaining patients, complete closure of the leak was accomplished without exception. All patients recuperated well. Stent removal was performed in 13 patients, on average 45 days after initial implantation. Following stent removal, the underlying mucosa showed neither necrosis nor severe morphological changes. However, mucosal hyperproliferation was visible at the stent margins. After stent removal, a tiny fistula with a diameter of approximately 3mm was detected in one case and sealed successfully with cyanoacrylate glue.

**Figure 1:**
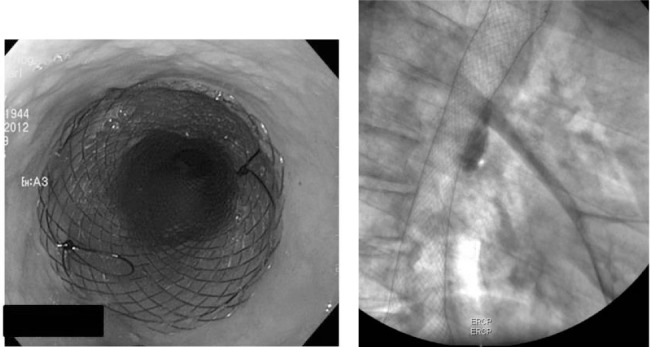
Endoscopic stent insertion for leakage of an oesophagogastrostomy: The endoscopic image (left) shows the stent already in place. The stent covers the anastomosis and seals the leak. The radioscopic image (right) shows the position of the stent in the mediastinum.

**Figure 2:**
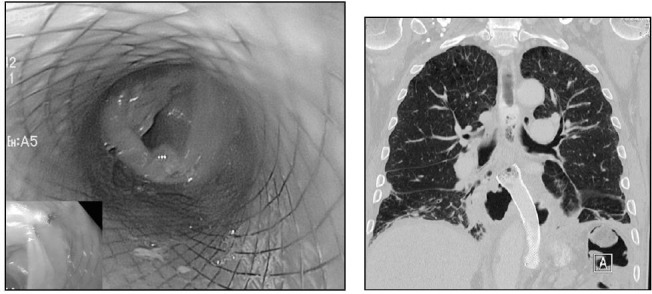
Endoscopic stent insertion for leakage of an oesophagojejunostomy: The endoscopic image (left) shows the already inserted stent and the intestinal mucosa of the jejunum is visible at the caudal end of the stent. The computed tomography (right) shows the stent in the inferior mediastinum. The stent’s position is much more distal than in Figure 1 because the oesophagojejunostomy is routinely performed inferior of the tracheal bifurcation.

### Statistical analysis

For analysis the study population was divided into two groups. Group A included all patients without leakage while Group B contained all cases showing an intrathoracic anastomotic leak. We statistically analysed the differences in age, frequency of neoadjuvant therapy, ASA grade and outcome between those two groups (Table 1).

There was no significant difference in age between the two groups. The mean age in Group A was 63.8 years while that in Group B was 64.8 years (*p*=0.54). Moreover, there was no difference in the frequency of neoadjuvant therapy (*p*=0.36) or the mean ASA grade (*p*=1.0). However, the risk for postoperative death was five times higher in patients with a leak (odds ratio: 5.10, 95% confidence interval: 2.06– 12.33, *p*<0.001).

## Discussion

Intrathoracic anastomotic leakage following oesophagectomy is a crushing complication. If proper drainage of the leak cannot be achieved, mortality can be up to 80%.^18^ Surgical re-exploration as well as simple conservative measures have shown discouraging results with fatal outcomes reported for between 40% and 100%.^1,2,6^ There is therefore an obvious need for new and more effective therapy strategies to reduce leak related mortality.

Endoscopic stent insertion has shown promising results in initial studies.^6–8^ Our own experience was also positive.^9^ For this reason, we changed our approach from surgical reexploration to endoscopic stent insertion in 2004. Following initial endoscopy to rule out ischaemia of the conduit or complete devastation of the anastomosis, endoscopic stent implantation has become our primary treatment option.

Although the initial findings were encouraging, it is well known that most disadvantages and risks are only revealed by long-term experience. The study period of eight years since introduction of endoscopic stent insertion as the primary treatment at our high volume centre allows us now to report on such long-term experience. The aim of this study was therefore to investigate the feasibility and results of endoscopic stent implantation as well as potential hazards and pitfalls.

Altogether we encountered 38 cases (13%) of intrathoracic anastomotic leak. This lies in the range reported for this complication.^2,19^ There were no differences in age, frequency of neoadjuvant therapy or ASA grade between patients with or without a leak. This implies that there were no substantial preoperative differences between patients who later sustained leakage and patients who had an uneventful postoperative course. Nevertheless, patients with a leak had a risk of mortality that was five times that of those without a leak. This highlights the importance of improved strategies and a more effective method for dealing with this life-threatening complication.

At first glance, the number of dehiscent anastomoses that could be managed by endoscopy seems rather disappointing. Despite being the preferred treatment option, stent implantation was only feasible in 22 cases while rethoracotomy was still unavoidable in 15 cases. The main reasons for surgical re-exploration were ischaemic gangrene of the pulled up gastric tube and advanced empyema requiring thorough debridement of the pleural cavity. This suggests that endoscopic stent insertion is the appropriate approach more often than not whereas there remains a considerable number of patients who still require surgical re-exploration. Earlier diagnosis of the leak might increase the number of patients suitable for endoscopic stent insertion by avoiding advanced pleural empyema. However, ischaemia of the conduit will continue to be the most common factor limiting the use of an endoscopic stent.

In our study, patients who required rethoracotomy had a risk of fatal outcome twice that of patients receiving endoscopic stent insertion (Table 2). However, the surgically reexplored leaks usually represented the worse cases, which were not suitable for endoscopy, and hence this result was to be expected. From the start, the two groups were highly incongruous regarding their prognosis and the observed outcome simply expresses this difference. Nevertheless, we achieved recovery in approximately 80% of the cases managed by endoscopic stenting. This compares very favourably with numbers reported thus far. Hitherto, conservative measures as well as operative treatment have generally been associated with mortality rates of between 40% and 100%.^1,2^

**Table 2:** Mortality for intrathoracic anastomotic leaks

Variables	All	Stent	Re-exploration
Total number of patients	38	22	15
Fatal outcome	12 (31%)	5 (22%)	6 (40%)
Odds ratio: 2.22
95% confidence interval: 0.43–12.16
*p*=0.30

Moreover, the long-term perspective of our study revealed two noteworthy phenomena, namely successful endoscopic management of American Society of Anesthesiology tracheo-oesophageal fistulas and the risk of American Society of Anesthesiology vascular erosion. Fistulisation between the airways and an insufficient intrathoracic anastomosis is a rare complication. Surgical repair has shown discouraging results. Consequently, the outcome had so far been mostly fatal due to persistent contamination of the tracheobronchial tree, causing pneumonia and, eventually, respiratory failure. We were able to manage five such cases by endoscopic stent insertion. The use of either a single tracheal stent, a single oesophageal stent or a combination of both provided an immediate end to the fistula and allowed recovery in four cases. This is a remarkable result that opens up a new possible indication for endoscopic stent insertion.

Until now, reports on American Society of Anesthesiology vascular erosion had been mostly on patients with benign or malign stenosis of the oesophagus who received endoscopic stent insertion with a palliative intent to reopen the passage for oral intake. Erosion of the stent through the oesophageal wall into the aortic arch has been reported after prolonged stent placement of several months as well as after quite a short placement of just a few days.^20–24^

In our series, stent related erosion of the thoracic aorta occurred in three cases. Aorto-oesophageal fistulas established themselves between the 16th and 41st day after stent implantation. Subsequent to bleeding with haematemesis, hypovolaemic shock with death from exsanguination followed rapidly. In all three cases, endoscopic management had been marred by repeated stent dislocation. Consequently, in cases when correct stent placement is not readily achievable, treatment options other than endoscopic stent implantation should be considered to prevent a situation where prolonged mediastinal inflammation and mechanical irritation by the stent may contribute to an increased risk for developing an aorto-oesophageal fistula.

## Conclusions

Our study demonstrates that endoscopic stent implantation is an effective and reliable treatment for intrathoracic anastomotic leakage. Nevertheless, surgical re-exploration is still unavoidable in a substantial number of cases. Furthermore, stent insertion shows encouraging results in the management of leak-induced fistulisation between the airways and the oesophagus. Stent-related vascular erosion is a deadly complication of inappropriate stent placement. Better prevention seems achievable by improved patient selection.
